# From implicit assumptions to explicit ethics: assessment of normative frameworks in digitalization recommendations in rheumatology

**DOI:** 10.3389/fdgth.2026.1928292

**Published:** 2026-10-21

**Authors:** Joanne Drexler, Jutta G. Richter, Rüdiger Buchkremer, Gamal Chehab, Birgitt Killersreiter, Christian Thielscher

**Affiliations:** 1 FOM University of Applied Sciences, Cologne, Germany; 2 Department of Rheumatology, University Hospital Düsseldorf, Medical Faculty of Heinrich-Heine-University, Düsseldorf, Germany; 3 Hiller Research Center, University Hospital Düsseldorf, Medical Faculty of Heinrich-Heine-University, Düsseldorf, Germany; 4 Center for Digital Medicine, University Hospital Düsseldorf, Medical Faculty of Heinrich-Heine-University, Düsseldorf, Germany; 5 Institute of IT Management and Digitization Research (ifid), FOM University of Applied Sciences, Düsseldorf, Germany; 6 Department of Rheumatology and Clinical Immunology, KEM Kliniken Essen-Mitte, Essen, Germany; 7 Competence Center for Medical Economics, FOM University of Applied Sciences, Cologne, Germany

**Keywords:** digitalization of medicine, ethical tensions, guidelines & recommendations, normative theories, rheumatology

## Abstract

**Background:**

The digital transformation of rheumatology offers significant potential but introduces complex ethical challenges.

**Objectives:**

This study investigates whether current guidelines and recommendations from leading international rheumatology societies provide a systematically reasoned ethical framework.

**Methods:**

We conducted a qualitative document analysis of digitalization-related publications from the American College of Rheumatology (ACR), Asia Pacific League of Associations for Rheumatology (APLAR), and European Alliance of Associations for Rheumatology (EULAR). A systematic search (2019–2025) identified five core documents. Using deductive content analysis guided by Beauchamp and Childress’ principlism and three major normative theories (utilitarianism, deontology, virtue ethics), we analyzed the presence and depth of ethical reasoning.

**Results:**

All documents acknowledge practical benefits and mention concepts like data protection and patient-centeredness. However, systematic engagement with normative ethical theories is absent. Ethical terms are used sporadically without definition, and fundamental tensions (e.g., efficiency vs. equitable access) remain invisible. A utilitarian perspective appears implicit in the prioritization of aggregate benefits, but this is not explicitly acknowledged or balanced against alternative frameworks.

**Discussion:**

Current digitalization guidelines in rheumatology exhibit a critical gap in explicit ethical scaffolding. To ensure responsible innovation, future guideline development should integrate multidisciplinary expertise and systematically apply diverse ethical frameworks, moving from implicit assumptions to transparent value deliberation.

## Introduction

The digitalization of medicine is fundamentally reshaping care: the integration of telemedicine, mobile health (mHealth) applications, wearable sensors, artificial intelligence (AI) and other technical developments offers significant potential to enhance long-term, complex care for patients; this is specifically important for patients with chronic disease, as in rheumatology (1, 2). These technologies could improve therapy adherence, enable continuous remote monitoring, and facilitate communication, ultimately aiming for more personalized and efficient care (3).

However, this technological shift is not merely a practical evolution; it is accompanied by a variety of ethical challenges, including (but not limited to) data security (4), transparency of decision-making (5), and the risk of a “digital divide” which threatens to exacerbate existing health inequalities, potentially excluding older, less technologically adept, or socially disadvantaged patient groups (6). These issues touch upon core ethical principles like autonomy, justice, beneficence, and non-maleficence (7). One aspect that is rarely considered in this context is that the disruptive potential of digitalization could also massively change collaboration in medicine, i.e., its “management"—including the authority to make decisions; this, too, can have significant ethical consequences (8).

Clinical guidelines and recommendations for optimal clinical care provided by professional societies are essential instruments for standardized care and translating evidence into practice. Their primary purpose is to support evidence-based clinical decision-making and, therefore, they are not designed to explicitly articulate or engage with philosophical theories or broader ethical considerations. With the increasing digitalization of healthcare, guidelines are increasingly expected to provide guidance not only on the appropriate use of digital technologies but also on how to responsibly navigate the ethical and societal challenges that arise from their implementations.

However, there is no such thing as one single ethical theory; rather, there are several theories, e.g., utilitarian, deontological, virtue ethical, to name just a few. As has been shown earlier by ethical decomposition, different normative theories can view moral problems differently, leading to distinct and sometimes conflicting recommendations; in the trolley case (an uncontrolled trolley is going to kill 5 persons on the track unless you throw a switch in which case it kills one person on another track), for example, a utilitarian would throw the switch whereas a Kantian would not (9). This is because utilitarian ethics hold that actions are right if they maximize happiness for the greatest number, even at the cost of an individual (there are several subtypes of utilitarianism which may treat this conflict differently). Kantianism, by contrast, centers on the individual’s moral autonomy and dignity, insisting that no one should be used merely as a means, even for the benefit of all. Aristotelian ethics seeks a balance, viewing the individual's flourishing (eudaimonia) as inherently tied to life within a community, where personal virtue contributes to the common good. All of this holds true for ethics in medicine as well. For example, a “utilitarian” guideline might prioritize efficiency at the potential expense of equality (a deontological concern), without even explicitly acknowledging this tension.

Therefore, it is paramount that guidelines (we use this term here as umbrella term for any type of recommendation, points to consider, etc.) are ethically reflective and transparent about their normative foundations. This study investigates to what extent the guidelines and comparable articles of leading international medical societies that comment about ethics of digitalization incorporate and discuss different ethical theories within the documents. We are aware that ethical consideration may have played a role in the guidelines development processes and might have been omitted in the published guidelines texts due to various reasons. We also analyze whether these documents provide a merely technical roadmap or a comprehensively reasoned ethical framework for the digital future of rheumatology. This study therefore asks: To what extent do current rheumatology guidelines on digital topics engage with normative ethical theories? And do they provide clinicians with the tools to navigate the value conflicts inherent in digital care?.

## Methods

### Study design and scope

We conducted a qualitative document analysis following established content analysis principles (10). The study was designed as a qualitative assessment rather than a systematic review, acknowledging that the aim was interpretive depth rather than exhaustive coverage.

### Document selection

We focused on three major international rheumatology societies—the American College of Rheumatology (ACR), the Asia Pacific League of Associations for Rheumatology (APLAR), and the European Alliance of Associations for Rheumatology (EULAR)—selected for their global influence and active role in developing practice guidance.

The screening process was conducted in two sequential steps. First, the identified literature was assessed to determine whether it addressed digitalization recommendations in Rheumatology. Second, the relevant literature was examined to determine whether and how ethical theories were considered. This two-step approach enabled us to distinguish between literature focusing on guidelines and literature that additionally engaged with ethical theory.

A systematic search was performed in PubMed and supplemented by a non-specific search engine (Google) to identify English-language publications from January 2019 to September 2025. The start date of 2019 was chosen to capture documents reflecting recent technological acceleration (including COVID-19-driven digital adoption).

Search terms for PubMed and Google included combinations of society names (EULAR, ACR, APLAR) with the keywords: “*digital*"*, “*artificial intelligence”* OR “*AI”*, “*telemedicine”*, “*mobile health”*, “*mHealth”*, “*rheumatology”*, and “*guidelines”* OR “*recommendations”* OR “*points to consider”* OR “*position statement”*.

Inclusion criteria were: (a) published or endorsed by ACR, APLAR, or EULAR; (b) primary focus on digital technologies in rheumatology; (c) document type constituting official guidance (guideline, points to consider, position statement). Exclusion criteria were: (a) conference abstracts; (b) editorials without society endorsement; (c) documents not addressing digital topics specifically.

After screening 202 titles and abstracts, and removing duplicates, five core publications were selected as the most current and comprehensive society-led guidance documents available: two from ACR (13, 14), two from EULAR (11, 12), and one from APLAR (15). These represent the full set of dedicated digitalization guidance documents from these societies during the study period.

### Analytical framework

We developed a deductive coding framework based on three pillars:

1. **Ethical Terms:** Presence of keywords such as autonomy, justice, beneficence, non-maleficence, informed consent, equity, data protection, privacy.
2. **Normative Theories:** Explicit reference to or implicit alignment with principlism (Beauchamp/Childress), utilitarianism (focus on aggregate outcomes), deontology/Kantianism (focus on duties, rights, individual dignity), or virtue ethics (focus on character and practical wisdom).
3. **Ethical Tensions:** Acknowledgment of trade-offs (e.g., efficiency vs. equity, privacy vs. innovation).

JD and CT coded the analyzed documents in accordance to the analytical framework. Formal intercoder agreement was assessed.

The analysis focused on whether ethical concepts were merely named or whether they were elaborated, defined, or situated within a theoretical framework.

## Results

### Overview of included documents

Table 1 provides a structured summary of the analyzed documents.

**Table 1:** Characteristics and ethical content of included documents.

Authors	Document Title	Year	Digital Domain	Development methodology	Target audience	Ethical Terms Defined?	Normative Theory Invoked?
de Thurah et al. (11)	EULAR points to consider for remote care in rheumatic and musculoskeletal diseases	2022	Remote Care	Developed by international Task Force, members invited from EULAR	Rheumatologists engaged in in- and outpatient care	No	No
Najm et al. (12)	EULAR points to consider for the development, evaluation and implementation of mobile health applications aiding self-management in people living with rheumatic and musculoskeletal diseases	2019	mHealth	Developed by international Task Force, members invited from EULAR	Rheumatologists engaged in in- and outpatient care, technical partners	No	No
ACR (13)	American College of Rheumatology Position Statement—Subject: Use of Artificial Intelligence in Rheumatology	2023	Artificial Intelligence	ACR Position statements are written by the Committee on Rheumatologic Care and approved by the ACR Board of Directors	Rheumatologists engaged in in- and outpatient care	No	No
ACR (14)	ACR 2024: American College of Rheumatology Position Statement—Subject: Telemedicine	2024	Telemedicine	ACR Position statements are written by the Committee on Rheumatologic Care and approved by the ACR Board of Directors	Rheumatologists engaged in in- and outpatient care	No	No
Ahmed et al. (15)	APLAR recommendations on the practice of telemedicine in rheumatology	2022	Telemedicine	Written by the Taskforce der Asia Pacific League of Associations for Rheumatology	Rheumatologists engaged in in- and outpatient care	No	No

EULAR, European Alliance of Associations for Rheumatology; ACR, American College of Rheumatology; APLAR, Asia Pacific League of Associations for Rheumatology.

### Ethical content

The key finding is a consistent pattern across all documents: while digitalization is welcomed for its practical benefits, systematic ethical reflection is peripheral.

#### Ethical terms

Concepts such as “data protection,” “privacy,” “patient-centeredness,” and “access” appear in all documents. However, they are used as catchwords without definition or elaboration. For example, the EULAR mHealth guideline (12) lists “user privacy and safety” as criteria but does not explore what privacy means in the context of third-party apps or how safety should be balanced against autonomy. The ACR AI statement (13) mentions “bias” and “confidentiality” but does not define these terms or discuss their ethical implications in depth.

#### Normative theories: no document explicitly references or utilizes any normative ethical theory

There is no mention of utilitarianism, deontology, virtue ethics, or principlism. The documents operate on a functional level, assuming the benefits of digitalization without situating these assumptions within a broader ethical framework.

#### Implicit utilitarianism

A pattern emerges across documents: recommendations prioritize system-level efficiency, population health benefits, and technological progress. While this emphasis on aggregate benefits aligns with a utilitarian logic (maximizing good for the greatest number), it is not explicitly acknowledged as such, and no counterbalancing deontological concerns (e.g., individual rights against algorithmic decisions, dignity in remote vs. face-to-face care) are explored. This interpretive hypothesis is supported by the consistent focus on outcomes and efficiency without parallel attention to potential harms to individual autonomy or justice.

#### Ethical tensions

Fundamental tensions remain undiscussed. For instance, the ACR telemedicine statement (14) notes regional “disparities” but does not frame this as a justice issue requiring trade-offs between expanding access and ensuring equitable distribution of resources. The APLAR document (15) mentions “structural hurdles” in the Asia-Pacific region but stops short of asking whether telemedicine might inadvertently exacerbate inequities for already marginalized populations.

Formal intercoder agreement did not show differences.

## Discussion

This qualitative assessment reveals a significant gap between technological advancement in rheumatology and the ethical scaffolding supporting its clinical implementation. The analyzed documents from ACR, APLAR, and EULAR function primarily as technical and operational manuals.

While the analyzed documents nod to ethical concepts, these remain undefined and ungrounded in normative theory. The absence of explicit engagement with frameworks like utilitarianism, deontology, or virtue ethics leaves guideline users without the conceptual tools to navigate value conflicts.

Our findings reflect the selected guidance documents rather than digital rheumatology guidance as a whole.

The absence of explicit references to ethical theories in the analyzed guidance documents should not be interpreted as indicating that ethical considerations were absent from the guideline development process. Rather, it is important to acknowledge that ethical considerations may be embedded implicitly within the recommendations, their underlying assumptions, and the priorities they establish. In fact, guidelines consistently address issues such as privacy, patient-centered care, transparency, bias and digital inequality, even if these are not explicitly framed within formal ethical theories. Our analysis focused on identifying and interpreting these implicit ethical dimensions rather than assessing the extent to which guidelines explicitly engage with ethical theories. In this context pragmatic considerations in guideline development, an emphasis on implementation, and editorial or space constraints might have hampered more explicit ethics related statements in the documents.

### Beyond implicit assumptions

The documents’ implicit utilitarian tilt—prioritizing aggregate benefits—is not inherently problematic. Indeed, improving population health outcomes is a legitimate and important goal. However, without explicit acknowledgment, this framework risks marginalizing other legitimate ethical concerns. As the trolley problem illustrates (9), deontology (16), utilitarianism (17), and virtue ethics (18) can yield conflicting recommendations which must be balanced for clinical recommendations (19). A guideline that implicitly adopts one framework without transparency denies stakeholders the opportunity to deliberate trade-offs.

Consider a concrete example: an AI tool that improves population-level outcomes by optimizing treatment allocation (a utilitarian gain) might rely on opaque algorithms that undermine individual autonomy and informed consent (a deontological concern). How should a clinician balance these competing values? Current guidance does not equip clinicians to answer this question. Similarly, a telemedicine program that increases overall access to care might inadvertently exclude elderly patients with limited digital literacy. The utilitarian calculus of “more care for more people” masks the deontological duty to ensure that no vulnerable group is left behind. Without explicit engagement with these frameworks, such tensions remain invisible and unaddressed.

### Contextualizing the findings: ethics beyond digitalization

It is important to stress that ethical dilemmas are not unique to digitalization. Physicians navigate value-laden decisions daily within healthcare systems—resource allocation, treatment prioritization, and access disparities long predate digital tools. For example, if data show that biologics are prescribed less frequently to patients beyond “working age,” this may reflect an implicit utilitarian logic (maximizing workforce contribution) operating beneath the surface of clinical decision-making. Digital technologies, however, can amplify and render visible these underlying ethical tensions. Algorithms can automate and scale such biases, making explicit ethical deliberation even more urgent. Thus, the call for ethical integration is not a critique of digitalization *per se*, but a recognition that digital tools act as force multipliers for existing value choices. They do not create ethical problems ex nihilo; rather, they make it impossible to ignore the ones that were always there.

### A collaborative note

The authors wish to emphasize that this analysis is not a criticism of the colleagues who have undertaken the difficult task of developing these guidelines. Ethical digitalization requires expertise in medicine, computer science, and philosophy—three complex fields that no single individual can master. The guideline developers whose work we analyzed have made valuable contributions to standardizing and advancing digital care. Our intent is to highlight an opportunity for enhancement, not to diminish existing efforts. The gap we identify is a systemic one, reflecting the novelty of digital ethics as a field and the challenges of interdisciplinary collaboration.

### Toward actionable recommendations

To bridge the identified gap, we propose practical “minimum ethics components” for future digital guidance:

1. **Explicit Value Deliberation:** Guidelines should include a dedicated ethics section that identifies key value tensions (e.g., efficiency vs. equity, privacy vs. innovation, individual autonomy vs. population health) and explains how trade-offs were weighed. This section should reference the normative frameworks that guided these deliberations.
2. **Stakeholder Impact Assessment:** Recommendations should be accompanied by an analysis of how they affect vulnerable groups (elderly, low-income, digitally excluded, non-native speakers) and propose specific mitigation strategies. This moves beyond acknowledging disparities to actively designing for equity.
3. **Multidisciplinary Development:** Working groups should routinely include ethicists, patient advocates, and social scientists alongside clinicians and technical experts. These team members should have equal standing in the development process, not merely consultative roles.

### Limitations

This study has several limitations that warrant consideration.

First, the document sample is small (*n* = 5) and restricted to English-language publications from three Western and Asia-Pacific societies. This limits generalizability to other specialties, other regions, and non-English guidance.

Second, the absence of explicit ethical theory does not necessarily imply the absence of ethical reasoning. It is possible that guideline developers applied principle-based ethics implicitly, without using theoretical labels. Our analysis captures only what is documented, not what may have occurred in internal deliberations.

Third, the interpretive claim of “implicit utilitarianism” is a hypothesis supported by textual patterns (emphasis on aggregate outcomes, efficiency, population-level benefits) but not directly verifiable. Alternative readings—for instance, that the documents reflect a pragmatic, problem-driven approach rather than any specific ethical framework—are possible and should be considered.

Fourth, the search strategy, while systematic, may have missed relevant documents. We focused on documents specifically about digitalization; broader society ethics statements that touch on digital topics peripherally were excluded. Future research should examine whether those documents fill the gap identified here.

Fifth, the analysis was limited to publicly available documents and did not include interviews with guideline developers, which might reveal ethical considerations that did not make it into the final text.

Future research should expand the scope to include other medical specialties, non-digitalization guidelines, non-English guidelines, and longitudinal comparisons to track whether ethical engagement increases over time. Qualitative interviews with guideline developers could also illuminate the barriers to integrating explicit ethical frameworks.

## Data Availability

The raw data supporting the conclusions of this article will be made available by the authors, without undue reservation.
